# Unique virulence role of post-translocational chaperone PrsA in shaping *Streptococcus pyogenes* secretome

**DOI:** 10.1080/21505594.2021.1982501

**Published:** 2021-10-01

**Authors:** Zhao-Yi Wu, Anaamika Campeau, Chao-Hsien Liu, David J. Gonzalez, Masaya Yamaguchi, Shigetada Kawabata, Chieh-Hsien Lu, Chian-Yu Lai, Hao-Chieh Chiu, Yung-Chi Chang

**Affiliations:** a Graduate Institute of Microbiology, College of Medicine, National Taiwan University, Taipei, Taiwan; b Department of Pharmacology and Skaggs School of Pharmacy and Pharmaceutical Sciences, University of California, San Diego, CA, USA; c Department of Oral and Molecular Microbiology, Osaka University Graduate School of Dentistry, Suita, Japan; d Department of Clinical Laboratory Sciences and Medical Biotechnology, College of Medicine, National Taiwan University, Taipei, Taiwan

**Keywords:** Parvulin-type peptidyl-prolyl isomerase, PrsA, Streptococcus pyogenes, secretome, virulence

## Abstract

*Streptococcus pyogenes* (group A *Streptococcus*, GAS) is a strict human pathogen causing a broad spectrum of diseases and a variety of autoimmune sequelae. The pathogenesis of GAS infection mostly relies on the production of an extensive network of cell wall-associated and secreted virulence proteins, such as adhesins, toxins, and exoenzymes. PrsA, the only extracellular parvulin-type peptidyl-prolyl isomerase expressed ubiquitously in Gram-positive bacteria, has been suggested to assist the folding and maturation of newly exported proteins to acquire their native conformation and activity. Two PrsA proteins, PrsA1 and PrsA2, have been identified in GAS, but the respective contribution of each PrsA in GAS pathogenesis remains largely unknown. By combining comparative proteomic and phenotypic analysis approaches, we demonstrate that both PrsA isoforms are required to maintain GAS proteome homeostasis and virulence-associated traits in a unique and overlapping manner. The inactivation of both PrsA in GAS caused remarkable impairment in biofilm formation, host adherence, infection-induced cytotoxicity, and *in vivo* virulence in a murine soft tissue infection model. The concordance of proteomic and phenotypic data clearly features the essential role of PrsA in GAS full virulence.

## Introduction

*Streptococcus pyogenes* (group A *Streptococcus*, GAS), a Gram-positive bacterium, is a strict human pathogen causing more than 700 million infections and 500,000 deaths annually [1]. GAS infection has very diverse clinical manifestations ranging from self-limiting superficial infection such as pharyngitis (strep throat) and impetigo to life-threatening invasive infection such as necrotizing fasciitis and streptococcal toxic shock syndrome (STSS). The ability of GAS to cause these distinct human infections is associated with the production of an extensive repertoire of cell wall-associated and secreted virulence proteins, such as M protein, pilus, IgG endopeptidase, C5a peptidase, Sda1 DNase, SpeB cysteine protease, pore-forming toxins, and superantigens. These factors exert their function additively or interdependently to facilitate optimal colonization, acquire essential nutrients, combat host defense responses, and promote invasion and spreading to deeper tissue [2].

In Gram-positive bacteria, these virulence factors are typically transported across the cell membrane by the secretory (Sec) translocase into the membrane-cell wall space in an unfolded state and are subsequently folded into their active form assisted by bacterial extracellular chaperone proteins [3,4]. Translocation of nascent polypeptides across the cellular membrane in GAS has been reported to occur at a discrete microdomain, ExPortal, located adjacent to the septum-forming area [5]. A surface interactome analysis revealed that several proteins involved in protein folding and transport mechanisms, such as OppA, DppA, PrsA, and HtrA, are colocalized in the ExPortal, and might be even part of the ExPortal complex [6]. This implies that the extracellular chaperones may be clustered in the ExPortal where the newly synthesized proteins can interact with chaperones essential for correct folding before secretion.

In Gram-positive bacteria, PrsA is the only parvulin-type peptidyl-prolyl *cis*/*trans*-isomerases (PPIase) involved in assisting protein folding and maturation outside the cytoplasmic membrane. It is a ubiquitous 30-kDa lipoprotein anchored to the outer leaflet of the cell membrane and localized in the space between the plasma membrane and cell wall to act as an extracellular chaperone to assist secreted proteins in acquiring their native conformation and activity [7]. The role of PrsA has been studied in several Gram-positive bacteria, such as *Bacillus subtilis, Listeria monocytogenes, Staphylococcus aureus, Streptococcus mutans,* and *Clostridioides difficile*, which demonstrated that PrsA plays an important role as a molecular chaperone of proteins involved in cell wall biogenesis [8,9], resistance to external stress and antibiotics [10–14] and bacterial virulence [8,9]. These observations suggest that PrsA significantly contributes to the pathogenesis of many disease-causing Gram-positive bacteria.

So far, *L. monocytogenes* and GAS are the only two Gram-positive bacterial species known to have two PrsA isoforms, PrsA1 and PrsA2 [15,16]. The importance of PrsA2 in listerial cell wall integrity, swimming motility and pathogenesis has been extensively studied, but PrsA1 was dispensable for all the tested biological functions [16–19]. GAS PrsA1 and PrsA2 lack the complete PPIase signature motif, which is critical for the propyl isomerization activity and shows low amino acid sequence similarity to listerial PrsA1 and PrsA2 [16], suggesting that GAS PrsA may function differently from listerial PrsA.

In this study, we combine comparative proteome and phenotype analysis to elucidate the role of PrsA in GAS physiology and pathogenesis. We showed that both PrsA1 and PrsA2 play unique and indispensable roles in shaping the GAS secretome and regulating GAS pathogenesis. GAS deficient in both *prsA1* and *prsA2* exhibited a remarkable loss of multiple virulence determinants and significant attenuation in biofilm formation, host adherence, and infection-induced cytotoxicity. Moreover, GAS Δ*prsA1/A2* double mutant is also less virulent in the murine infection model, supporting the important role of PrsA in GAS disease progression *in vivo*.

## Results

### Characterization of the prsA deletion mutant

**Figure 1.**
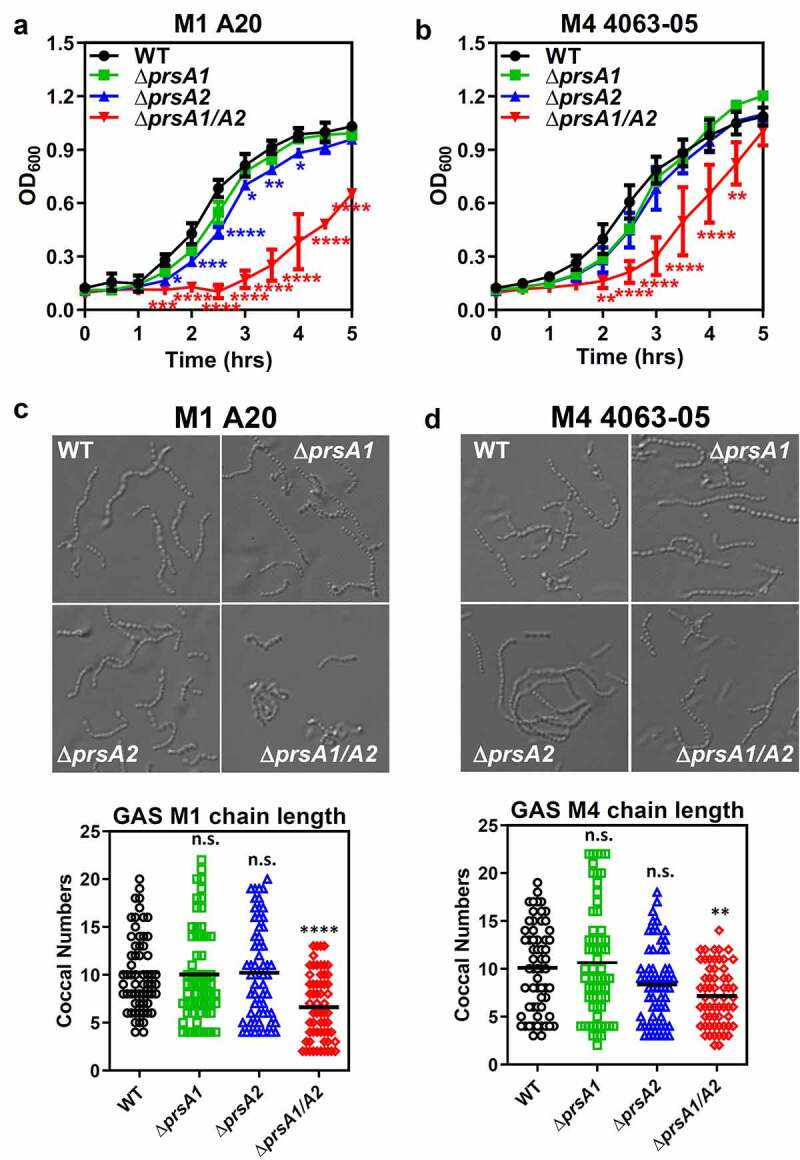
Growth characteristics and morphology of GAS WT and *prsA* deletion mutants. The overnight-grown GAS WT and Δ*prsA* mutants generated in the M1 strain A20 (a) or M4 strain 4063–05 (b) were diluted with fresh THY to OD_600_ of 0.1. Aliquots were taken every 30 min and the optical density was read with a spectrophotometer at 600 nm. Data shown were mean ± SD pooled from two independent experiments. Statistical significance was determined in comparison to WT by unpaired *t* test. Bright-field imaging of stationary phase M1 WT and its *prsA* deletion derivatives (c) and M4 WT and its *prsA* deletion derivatives (d). To determine bacterial chain lengths, 60 chains were randomly selected and counted. Statistical significance was determined in comparison to WT by one-way ANOVA multiple comparison test. *, *P*< 0.05; **, *P*< 0.01; ***, *P*< 0.001; ****, *P*< 0.0001

PrsA2 has been shown to play a critical role in the final maturation step of SpeB in M1 GAS [20]. To further explore the functional contribution of individual PrsA isoforms in GAS physiology, isogenic mutants lacking the *prsA1, prsA2* or both *prsA1* and *prsA2* were generated in the invasive encapsulated M1 GAS by in-frame allelic replacement. Genes neighboring *prsA1* and *prsA2* are shown in Figure S1(a). Replacement of the *prsA* gene in the GAS chromosome with the antibiotic selection marker, *cat* gene, was confirmed by PCR (Figure S1(b)). Mutants lacking single *prsA* gene (M1Δ*prsA1* and M1Δ*prsA2*) showed comparable growth kinetics to WT, though M1Δ*prsA2* mutant showed moderate growth impairment (Figure 1(a)). Loss of both *prsA* genes (M1Δ*prsA1/A2*) resulted in severe growth attenuation and chain length shortening (Figure 1(a,c)). Although this observation indicates that PrsA exerts critical roles in GAS physiology, the seriously decreased growth rate of M1Δ*prsA1/A2* at the same time limits its application in further functional assays. We next generated *prsA* deletion mutants in the M4 GAS, which is one of the major nonencapsulated serotypes responsible for mucosal and invasive GAS infections [21–24]. Although the chain length of M4 Δ*prsA1/A2* was also shorter than M4 WT, M4Δ*psA1/A2* mutant only showed delayed growth in the early log phase and was similar to M4 WT in the stationary phase (Figure 1(b,d)). The M4 *prsA* mutants did not display severe morphological defects such as kinks or twist in the chains in the scanning electron microscopy analysis, despite the coccal size of *prsA* mutants is slightly rounder and smaller than the WT (Figure S1(c)). Transmission electron microscopy analysis also did not reveal severe morphological defects in the *prsA* mutants besides the rounder shape of the *prsA* mutants (Figure S1(d)). Given that deletion of *prsA* in the M1 GAS background shows severe growth attenuation, we proceeded with the M4 GAS serotype for most of the following experiments.

### Deletion of prsA drastically altered the GAS secretome

**Figure 2.**
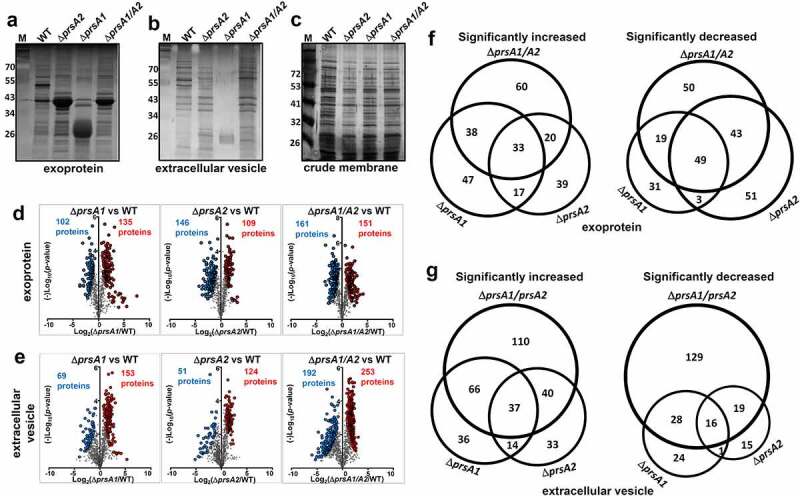
Quantitative proteomic analysis of M4 GAS WT and *prsA* deletion mutant. SDS-PAGE and silver stain analysis of M4 GAS proteins collected from cell-free culture media (a), extracellular vesicles (b) and crude membrane extracts (c). Differentially expressed proteins identified by the TMT proteome analysis were presented as a volcano plot depicting mean quantitation intensity ratios of the Δ*prsA* mutant versus WT plotted against logarithmic *t* test *P* values from 2 to3 biological experiments of each strain. Proteins with π > 2.5 were highlighted in red and blue to indicate upregulation and downregulation, respectively. Proteins with significantly altered abundance between WT and Δ*prsA* mutants were shown in (d) and (e) for exoproteome and EV proteome, respectively. Overview of significantly differential expressed exoproteins (f) and EV proteins (g) identified in Δ*prsA1*, Δ*prsA2* and Δ*prsA1/A2* mutants. Numbers of proteins exclusively detected in each sample or shared between them were indicated in the Venn diagrams

Given that PrsA has been suggested to assist extracellular protein folding in several Gram-positive bacteria [7], cell-free culture supernatants, secreted extracellular vesicles (EVs), and crude membrane extracts were collected for SDS-PAGE and silver stain analysis to study the role of PrsA in GAS protein stability and folding. Compared to WT M4 GAS, deletion of either *prsA* or in combination resulted in drastic alteration of the protein profile in culture supernatant (Figure 2(a)) and EVs (Figure 2(b)). Notably, a distinct protein banding pattern was found in Δ*prsA1* which is different from that in M4Δ*prsA2* and M4Δ*prsA1/A2* whose patterns are relatively similar. In contrast, the membrane protein profile between WT and *prsA* deletion mutants was broadly similar in protein banding pattern (Figure 2(c)). In order to determine whether deletion of *prsA* also affects the exoprotein profile in the M1 GAS background, we analyzed the cell-free culture supernatants collected from WT M1 GAS and its isogenic *prsA* deletion mutants. As shown in Figure S3(a), drastic alteration of the protein profile was observed in the culture supernatants collected from M1Δ*prsA2* and M1Δ*prsA1/A2* mutants, while the protein banding profile was largely similar between WT and M1Δ*prsA1* mutant. This observation suggests that PrsA1 and PrsA2 may affect its client proteins differentially expressed in serologically different strains.

To better understand how PrsA shapes the M4 GAS proteome, proteins collected from culture supernatants and EVs were subjected to quantitative TMT proteome analysis. There were 792 and 1264 proteins, respectively, quantified in our exoprotein and EV protein preparations, accounting over 40% of the open-reading frames (ORFs) encoded by the M4 GAS reference genome MGAS10750. Hierarchical clustering of the identified proteins shows that our biological replicates are highly positively correlated (Figure S2(a,b)). The identified protein list along with the normalized spectral counts and annotations is presented in Table S2 (exoproteome) and Table S3 (EV proteome).

Fold‐changes in protein scores relative to the WT and corresponding *p*‐values were revealed in the volcano plots to give a global overview of pairwise comparisons between WT and *prsA* deletion mutants. Exoproteins with significantly altered abundance in M4Δ*prsA1*, M4Δ*prsA2* and M4Δ*prsA1/A2* were, respectively, 135, 109 and 151 proteins upregulated, and 102, 146 and 161 proteins downregulated (Figure 2(d)). EV proteins with significantly altered abundance in M4Δ*prsA1*, M4Δ*prsA2* and M4Δ*prsA1/A2* were, respectively, 153, 124 and 253 proteins upregulated and 69, 51 and 192 proteins downregulated (Figure 2(e)). The numbers and representatives of unique and shared proteins with altered abundance identified between WT and *prsA* deletion mutants were illustrated by Venn diagrams to show the respective contribution of each individual PrsA isoform or in combination in shaping the GAS secretome (Figure 2(f,g)). A complete list of proteins with significantly altered abundance in *prsA* deficient mutants is shown in Tables S4–S7. At least 50% of these significantly changed proteins were only observed in M4Δ*prsA1* but not M4Δ*prsA2* mutant and vice versa. The overlapping yet distinct phenotypic changes in the exoproteome and the EV proteome of M4Δ*prsA1*, M4Δ*prsA2* and M4Δ*prsA1/A2* mutants suggest that both PrsA1 and PrsA2 play unique and indispensable roles in shaping the M4 GAS secretome, which is different from the reported negligible role of PrsA1 in listerial physiology and pathogenesis [17,25].

In order to elucidate the role of PrsA in GAS biology, proteins significantly different from WT in M4Δ*prsA1/A2* mutant (π > 2.5) were further analyzed by Kyoto Encyclopedia of Genes and Genomes (KEGG) pathway analysis. Ten and 19 KEGG pathways were identified with statistical significance (corrected *p* < 0.05 and contain more than 5 protein samples) in exoproteome and EV proteome, respectively. A majority of identified proteins were proteins associated with vital biological processes such as protein synthesis, purine and pyrimidine synthesis, pyruvate metabolism, DNA replication, protein export, and peptidoglycan biosynthesis (Tables 2 and 3).

**Table 1.** Bacterial strains and plasmids used in this study

Strain/Plasmid	Relevant characteristics*	Source
**Bacterial strains**		
A20	*Streptococcus pyogenes* M1T1 strain	[40]
M1Δ*prsA1*	*prsA1* deletion mutant in A20	This study
M1Δ*prsA2*	*prsA2* deletion mutant in A20	This study
M1Δ*prsA1/A2*	*prsA1*/*prsA2* double deletion mutant in A20	This study
4063-05	*Streptococcus pyogenes* M4 strain	[39]
M4Δ*prsA1*	*prsA1* deletion mutant in 4063-05	This study
M4Δ*prsA2*	*prsA2* deletion mutant in 4063-05	This study
M4Δ*prsA1/A2*	*prsA1*/*prsA2* double deletion mutant in 4063-05	This study
CΔ*prsA1*	*prsA1 trans*-complemented strain of M4Δ*prsA1*	This study
CΔ*prsA2*	*prsA2 trans*-complemented strain of M4Δ*prsA2*	This study
**Plasmids**		
pHY304	Temperature-sensitive shuttle vector, Erm^R^	[42]
pHY-prsA1	pHY304 + *prsA1* knockout construct (for M1 & M4)	This study
pHY-M1prsA2	pHY304 + *prsA2* knockout construct (for M1)	This study
pHY-M4prsA2	pHY304+ *prsA2* knockout construct (for M4)	This study
pLZ12Km2-P23R-TA	Complementation vector, Kan^R^	(52)
pLZ-prsA1	pLZ12Km2-P23R-TA+*prsA1* expression construct	This study
pLZ-prsA2	pLZ12Km2-P23R-TA+*prsA2* expression construct	This study
pET15b-prsA1	pET15b + *prsA1* expression construct	This study
pET15b-prsA2	pET15b + *prsA2* expression construct	This study

*Erm^R^, erythromycin resistance; Kan^R^, kanamycin resistance.

**Table 2.** KEGG pathway analysis for exoproteome

Term in KEGG pathway	Corrected *p*-value
**Upregulated in Δ*prsA1/A2* mutant**	
Aminoacyl-tRNA biosynthesis AsnS, CysS, GatB, HisS, LysS, MetS, PheT	3.2E-3
Protein export Ffh, SecA, SecY, SipC, Spi	9.9E-3
ABC transporter AdcC, EcfA2, FtsE, OppB, OpuAA, OpuABC, PstB1, PstS	1.2E-2
Peptidoglycan biosynthesis DacA1, Pbp1A, Pbp1B, Pbp2A, MurZ	1.2E-2
Ribosome RplC, RplD, RplS, RplW, RpsC, RpsD, RpsL	2.7E-2
**Dnregulated in Δ*prsA1/A2* mutant**	
Glycolysis/glyconeogenesis AcoL, AdhA, Eno, Fba, GapN, GlcK, Ldh, Pfk, Pgi, Pgk, Plr, Spy1342	2.2E-12
Pentose phosphate pathway DeoB, Fba, MipB, Pfk, Pgi, PgmA, Prs, PrsA2^#^, Spy1342, Ta1, Tkt	1.5E-10
Fructose and mannose metabolism Fba, FruA, Pfk, PgmA, PtsB, Spy1342, TpiA	5.3E-5
Purine metabolism Adk, ArcC, DeoB, DeoD, DnaN, GuaA, NrdF, PrsA2^#^, Spy0996, Spy0998	1.8E-4
Pyrimidine metabolism ComEB, DeoD, DnaN, NrdF, PyrC, Udp, Spy1469, Upp	3.4E-3

*Pathways with corrected *p*-value less than 0.05 and involved more than five protein numbers are considered significant.

^#^ribose-phosphate pyrophosphokinase

**Table 3.** KEGG pathway analysis for EV proteome

Term in KEGG pathway	Corrected *p*-value
**Upregulated in Δ*prsA1/A2* mutant**	
Aminoacyl-tRNA biosynthesis AlaS, ArgS, AsnC, AspS, GatA, GatB, GltX, GlyS, HisS, LysS, MetS, Fmt, PheT, SerS, TrsA	3.8E-11
Mismatch repair DnaE, DnaX, HolA, LigA, MutL, MutS, MutS2, PolC, PcrA, RecJ Spy1212	2.9E-8
Pyrimidine metabolism CarA, CarB, DnaE, DnaX, HolA, NrdD, NrdE1, NrdE2, PolC, PyrB, PyrC, PyrH, Spy1212, ThyA, Udk	3.0E-7
Purine metabolism DnaE, DnaX, GuaB, GuaC, HolA, NrdD, NrdE1, NrdE2, PurA, PolC, Pyk, RelA, Spy0298, Spy1212, Xpt	1.5E-6
DNA replication DnaE, DnaG, DnaX, HolA, HolB, LigA, PolC, Spy1212	3.8E-5
Pyruvate metabolism AccC, AckA, AcoA, AcoB, OadA2, PflD, Ppc, Pyk, Spy0041	3.3E-4
Ribosome RpsC, RplA, RplB, RplC, RplD, RplF, RplJ, RplO, RplU, RplV, RplW	6.2E-4
Alanine, aspartate and glutamate metabolism AsnA, CarA, CarB, GlmS, PurA, PyrB	6.9E-4
Selenoamino acid metabolism MetB, MetS, Meth1, Metk2, Spy0261, Spy1106	1.5E-3
Butanoate metabolism AcoA, AcoB, PflD, MvaS1, Spy0041, Spy0553	5.0E-3
Peptidoglycan biosynthesis MurA, MurC, MurD, MurE, MurF, MurZ	1.3E-2
**Dnregulated in Δ*prsA1/A2* mutant**	
Fructose and mannose metabolism FruA, ManM, ManN, Pfk, PtsB, PtsC, PtsD, Spy0438, Spy1342	2.2E-6
Phosphotransferase system (PTS) FruA, AgaD,, ManM, ManN, LacE, PtsB, PtsC, PtsD, ScrA, Spy1512	3.1E-5
Arginine and proline metabolism ArcA, ArcB, ArcC, GlnA, Spy0941, Spy1053, Spy1378	1.7E-4
ABC transporters MalC, MalD, OppB, OppC, PotB, SagG, SagI, SrtF, Spy0624, Spy0980	2.4E-4
Pentose phosphate pathway Pfk, Pgi, Rpe, Tkt, Spy0996, Spy1342, Spy1487	2.7E-4
Amino sugar and nucleotide sugar metabolism GlcK, ManM, ManN, Pgi, PtsB, PtsC, PtsD, Spy1342	2.9E-4
Glycolysis/Gluconeogenesis Eno, GapN, GlcK, Pfk, Pgi, Plr, Spy1342	2.3E-3
Galactose metabolism AgaD, GlcK, LacE, Pfk, Spy1342, Spy1512	1.1E-2

*Pathways with corrected *p*-value less than 0.05 and involved more than five protein numbers are considered significant.

### Loss of prsA is associated with dysregulated abundance of many GAS virulence factors

**Figure 3.**
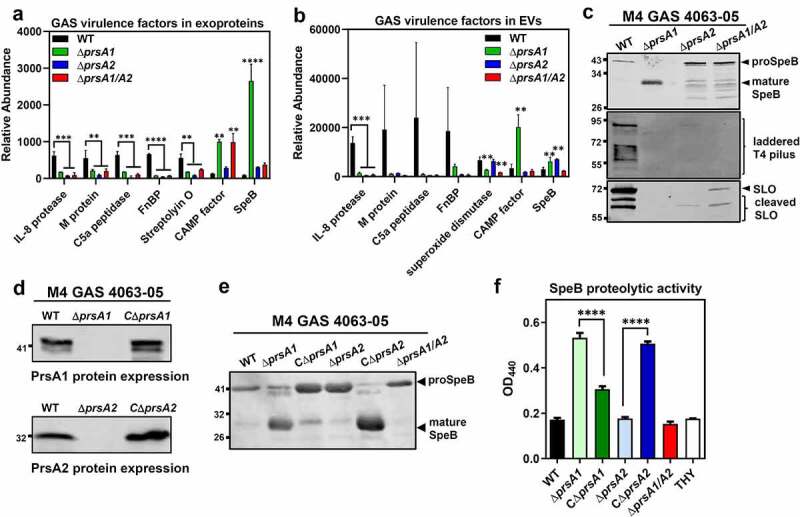
Dysregulated expression of GAS virulence factors in *prsA* deletion mutants. Representative demonstration of significantly altered virulence factors identified in M4 GAS exoproteome (a) and EV proteome (b). (c) Expression of SpeB, T4 pilus and SLO in the cell-free M4 GAS culture supernatant by Western blot analysis. (d) Expression of PrsA in the *prsA*-complemented M4 GAS strains. The crude membrane fractions were collected from WT, *prsA*-deficient mutants and *prsA*-complemented strains and the expression of PrsA proteins was analyzed by Western blot analysis with anti-PrsA1 and PrsA2 antibodies. (e) Expression and maturation of SpeB in the *prsA*-complemented M4 GAS strains. SpeB in the cell-free culture supernatant collected from stationary bacterial cultures were analyzed by Western blot analysis with anti-SpeB antibodies. (f) SpeB-mediated proteolytic activities. Filtered culture media were collected from stationary phase M4 GAS and incubated with 1% azocasein solution. The amount of cleaved azopeptides were determined by measuring the solution absorbance at 440 nm. Data shown were mean ± SD pooled from two independent experiments performed with biological triplicates. Statistical significance was determined in comparison to WT by one-way ANOVA multiple comparisons test (a, b, f). **, *P*< 0.01; ***, *P*< 0.001; ****, *P*< 0.0001

Pronounced alterations of secreted virulence factors were also revealed in our proteomic analysis, where more than 20 well-recognized GAS virulence factors are remarkably dysregulated in M4Δ*prsA1/A2* mutant (Tables 4 and 5). Of these, proteins associated with GAS adherence, such as fibronectin-binding proteins (FnBP), M proteins and pilus proteins, were drastically reduced in M4Δ*prsA1/A2* mutant. Proteases known to avoid complement activation and neutrophil recruitment, such as C5a peptidase (ScpA), oligoendopeptidase O (PepO), and IL-8 protease (SpyCEP) were also significantly diminished in M4Δ*prsA1/A2* culture supernatants. Moreover, proteins acting on detoxification of oxidative stress and induction of host cell death, such as peroxiredoxin (AhpC), NADH oxidase (NOX), superoxide dismutase (SodA) and streptolysin O (SLO) were also reduced in the absence of PrsA. In contrast, the abundance of CAMP factors, cysteine protease SpeB, and immunogenic secreted proteins Isp and Isp2 was increased in M4Δ*prsA1/A2* mutant. Relative abundance of selected virulence factors found in cell-free culture supernatants and EVs across WT and various *prsA* deletion mutants are shown in Figure 3(a,b), respectively.

**Table 4.** Virulence factors with altered abundance in Δ*prsA1/A2* culture medium

Downregulated	Bacterial adhesion and spread
	Fibronectin binding proteins: Spy1806, Spy1822, Spy1823 Pilus component: Spy0115, Spy0117
	**Immunomodulation and stress responses**
	ScpA, SpyCEP, Mac-1, PepO, AhpC, Spd, Spd3, Plr, GapN, Pgk, Hyl, Nga, SLO, SpeA2, Nox, Irr, CpsY
Upregulated	CAMP factor, Isp, Isp-2, Rgg, SibA, SpeB, CovS, LytR, RofA, Rgg

**Table 5.** Virulence factors with altered abundance in Δ*prsA1/A2* EV

Downregulated	Bacterial adhesion and spread
	Fibronectin binding proteins: Spy0114, Spy1822, Spy1823 Pilus component: Spy0115, Spy0116, Spy0117
	**Immunomodulation and stress responses**
	ScpA, SpyCEP, M protein, Mac-1, PepO, Spd3, Plr, GapN, Hyl, HylA, Nga, SpeA2, SodA
Upregulated	CAMP factor, Isp, Isp-2, SibA, CovS

In order to functionally validate our proteomic observations attributed by *prsA* deletion, we compared the amount and activity of several well-known virulence factors between WT and *prsA* deletion strains. Analysis of the culture supernatants by Western blot indicated that deletion of *prsA1* in M4 GAS results in increased amount of mature SpeB (28 kDa) which was barely detectable in the WT, while reduced maturation of SpeB was clearly observed in the M4Δ*prsA2* and M4Δ*prsA1/A2* mutant (Figure 3(c), upper panel). This impaired SpeB maturation was also observed in the M1Δ*prsA2* and M1Δ*prsA1/A2* mutant (Figure S3(b)), which is in consistent with the previously reported role of PrsA2 in SpeB maturation [20]. In addition to SpeB, the reduced abundance of pilus and SLO in M4Δ*prsA* culture supernatants was also confirmed by Western blot analysis (Figure 3(c), middle and lower panel), corroborating the TMT proteomic analysis.

Genetic complementation of the *prsA* deficient mutant with a plasmid expressing the intact *prsA1* and *prsA2* under constitutive promoter, respectively, restored the production of the PrsA1 and PrsA2 proteins in *prsA* deletion mutants (Figures S3(c) and 3(d)). In addition, complementation of Δ*prsA2* mutants with *prsA2* successfully rescued the SpeB maturation (Figures 3(e) and S3(d)) and proteolytic activity (Figures 3(f) and S3(e)) both in M1 and M4 GAS serotypes. In contrast, expression of *prsA1* in M1Δ*prsA1* and M4Δ*prsA1* mutants resulted in reduced SpeB maturation in M1 and M4 GAS, suggesting that PrsA1 may have a negative role in SpeB maturation. This finding was in line with the observation that deletion of *prsA1* in M1 GAS whose endogenous PrsA1 protein abundance was very low had a negligible role in SpeB maturation (Figure S3(b,c)) while deletion of *prsA1* in M4 GAS which has high PrsA1 protein levels caused drastically increased mature SpeB proteins. Together, our proteomic and functional observations indicate that PrsA may affect the abundance and/or activity of numerous GAS virulence determinants in varying degree depending on endogenous PrsA protein levels or the strain features.

### *PrsA is required for GAS biofilm formation, HaCaT keratinocyte adherence, and* in vivo *soft-tissue infection*

**Figure 4.**
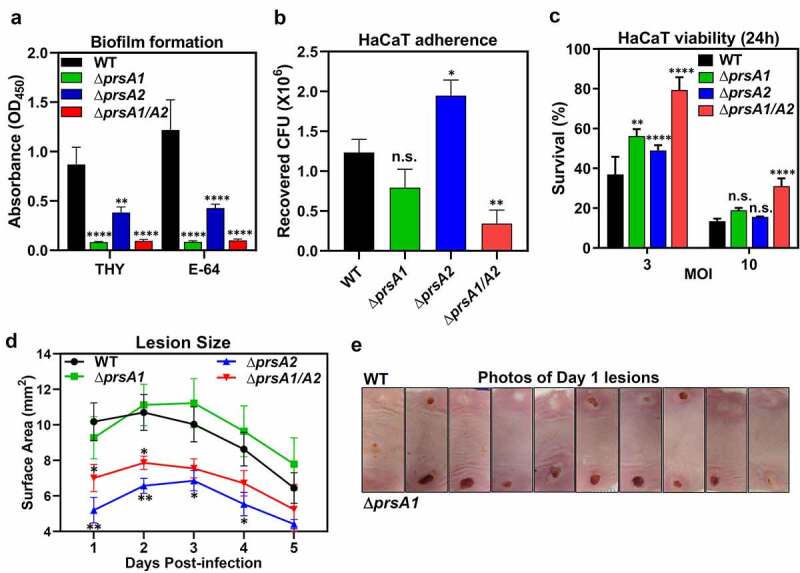
GAS full virulence was impaired in *prsA* deletion mutants. (a) Biofilm formation was impaired in *prsA* deletion mutants. M4 GAS was grown in 96-well polystyrene plates for 24 h in the presence or absence of SpeB inhibitor E-64, and the produced biofilm mass was stained with crystal violet and quantified by measuring the solution absorbance at 540 nm. (b) HaCaT cells were infected with M4 GAS at MOI of 10 for 30 min, followed by extensive wash to remove unbound GAS. Cell-associated GAS were enumerated by serial plating. (c) GAS-induced cell death was assessed by crystal violet staining of surviving HaCaT cells 24 h post M4 GAS infection. Data shown were mean ± SD pooled from at least two independent experiments performed with biological triplicates (a-c). (d) Lesion sizes from WT M4 GAS- and *prsA* deletion mutants-infected mice. Representative results from two independent experiments were shown as mean ± SD. (e) Photographs of necrotic skin lesions at day 1 from WT- and Δ*prsA1*-infected mice. Statistical significance was determined in comparison to WT by one-way ANOVA multiple comparisons test (a-c) or Mann–Whitney test (d). n.s., non-significant; *, *P*< 0.05; **, *P*< 0.01; ***, *P*< 0.001; ****, *P*< 0.0001

The ability of GAS to cause infection relies heavily on the production of an extensive network of cell surface and secreted exoproteins, such as M protein, pilus, C5a peptidase, Sda1 DNase, SpeB cysteine protease, and pore-forming toxins to facilitate host colonization and thwart host defenses [2]. We have shown that deletion of *prsA* gene causes aberrant expression of a wide variety of GAS virulence factors; thus, it was of interest to determine whether this observation is related to GAS pathogenesis. As many drastically reduced proteins in the Δ*prsA1/A2* mutant, such as fibronectin-binding proteins and pilus constituents, were associated with biofilm formation and cell adherence, we first investigated the effects of PrsA on these phenotypes. Biofilm formation by each GAS strain was quantified by crystal violet assay. The strongest biofilm formation in the crystal violet assay was observed for the WT, while Δ*prsA1*, Δ*prsA2*, and Δ*prsA1/A2* mutants all demonstrated significant reduction in the formation of biofilms even in the presence of SpeB inhibitor E-64 (Figure 4(a)). WT and *prsA* deletion mutants were next compared for their adherence phenotype on human HaCaT keratinocytes, presenting human superficial skin which is one of the major GAS infection sites. Adherence of Δ*prsA1/A2* mutant to HaCaT cells was significantly reduced, whereas similar and increased HaCaT adherence was observed for Δ*prsA1* and Δ*prsA2*, respectively, as compared with WT GAS (Figure 4(b)). Although *prsA* deletion mutants exhibited differential cell adherent capability, they were all less virulent in triggering the cell death of the infected HaCaT cells (Figure 4(c)). Together, our results indicate that PrsA affects proteins participate in bacterial adherence and subsequent infection-induced cell death.

We then examined the *in vivo* virulence of WT and Δ*prsA* mutants in a murine soft-tissue infection model, which resembles human necrotizing fasciitis. Animals were infected with either WT or Δ*prsA* mutants in opposing flanks, and lesion sizes were monitored daily. Infection with the Δ*prsA2 and* Δ*prsA1/A2* mutants resulted in significantly smaller lesions than the WT strain and Δ*prsA1* (Figure 4(d)). Despite similar lesion sizes being observed in mice infected with WT and Δ*prsA1*, Δ*prsA1* mutant induced lesion formation more rapidly than WT (Figure 4(e)). These observations suggest that both PrsA1 and PrsA2 play important roles in GAS virulence *in vivo*, although they may act in different directions. Together, our results indicate that GAS PrsA1 and PrsA2 play unique and indispensable roles in shaping the GAS secretome. Deletion of single or both *prsA* genes resulted in significantly dysregulated expression of many GAS virulence factors which are required for GAS full virulence.

## Discussion

GAS is genetically diverse, and the existence of discrete GAS population associated with different clinical manifestations and preferred tissue site of infection has long been recognized [26]. To understand the respective contribution of PrsA1 and PrsA2 in GAS physiology and pathogenesis, single and double *prsA* deletion mutants were generated in the most prevalent encapsulated M1 GAS and recently emerging nonencapsulated M4 GAS strains. The *prsA* double mutant in M1 GAS (M1Δ*prsA1/A2*) showed significant growth attenuation and increased cell death in the broth culture, while the M4Δ*prsA1/A2* showed moderate growth delay and subtle morphological changes compared to WT (Figure 1). Although M1 PrsA1 and PrsA2 are, respectively, 100% amino acid sequence identical to M4 PrsA1 and PrsA2, it affects their growth and morphology in varying degrees. The possible explanation for this discrepancy is the strain feature which is determined by bacterial proteins, e.g. surface antigens and virulence factors, expressed in serologically different strains. Therefore, the strain-specific phenotypes caused by *prsA* deletion could be attributed to the strain-specific client proteins of PrsA or varying protein abundance of PrsA expressing in different GAS strains. Similar strain-specific roles for PrsA-like proteins were observed in *Streptococcus pneumoniae* whose PpmA, the PrsA homologue, contributes to pneumococcal host colonization and phagocytic evasion in a strain-specific manner [27].

The PrsA-like proteins are formed by the N-terminal, C-terminal and center PPIase domains. PrsA-like proteins identified from most of the Gram-positive bacterial species exhibit the complete PPIase signature motif except those from the *Streptococcaceae* family whose PPIase domain is defective in the key amino acids known to be critical for PPIase activity [15,16]. Despite having this intrinsic difference, a profound impairment in protein export, morphology, physiology, and virulence upon *prsA* deletion has been widely demonstrated in the streptococcal and lactococcal species [8,17,18,20,27–34]. Proteomic analyses have been conducted in *B. subtilis, L. monocytogenes*, and *S. aureus* whose PrsA is competent for PPIase activity to identify the potential substrate or interacting partner responsible for the phenotypic changes observed in the *prsA* deletion mutants [17,28,35]. However, this information is not available for most of the bacteria belonging to the *Streptococcaceae* family. To comprehensively study the proteomic changes in the absence of PrsA, we applied the TMT analysis to simultaneously quantitate protein abundance from samples collected from WT GAS or *prsA* deletion mutants. As shown in Figure 2, a significant change in the extracellular proteome was observed in GAS *prsA* deletion mutants, and more than 50% of these significantly changed proteins were only observed in Δ*prsA1* but not Δ*prsA2* mutant and vice versa. For example, previously identified PrsA2-interacting proteins, such as ABC transporter OppA and DppA and thiol disulfide isomerase TlpA [6], were significantly reduced in the exproteome of Δ*prsA2* mutant, whereas these proteins were either not changed or even upregulated in the exoproteome of Δ*prsA1* mutant (Table S2). This observation again supports the unique and indispensable role of each PrsA in GAS biology. In addition to the central PPIase domain, the N- and C-terminal domains of PrsA have been suggested to play a role in substrate selectivity in *L. monocytogenes* and *B. bacillus* [18,19,36]. There is approximately 65% amino acid sequence identity between GAS PrsA1 and PrsA2 (Figure S2(c)). The extra 40 amino acids in the C-terminal domain of PrsA1 possibly contribute, at least in part, to the overlapping and distinct proteomic changes observed in the GAS *prsA* single deletion mutant.

GAS is able to produce an arsenal of virulence factors for cell adhesion, cytotoxicity and immune evasion to establish a successful infection. The proteomic data revealed that substantial numbers of virulence-related proteins are drastically dysregulated in the absence of PrsA1 and PrsA2, such as M protein which interferes phagocytosis, IL-8 protease which blocks neutrophil chemotaxis to infectious sites, C5a protease which inhibits immune cell migration, and CAMP factor which promotes streptococcal host adhesion and invasion (Figure 3(a,b)). Consistent with the observed proteomic changes, biofilm formation, cell cytotoxicity, and *in vivo* murine virulence were also significantly impaired in the Δ*prsA1/A2* mutant (Figure 4). It was noted that the maturation of SpeB is oppositely regulated by PrsA1 and PrsA2 (Figure 3(a,c)). SpeB has been demonstrated to drastically degrade the GAS secretome [37], whereby proteins identified in this study showing different abundance in WT and Δ*prsA* mutants may not be attributed directly from PrsA chaperone activity but from the different SpeB activity. Although we cannot exclude the potential SpeB-mediated confounding effects associated with *prsA* deletion, we were still able to identify proteins, including important virulence factors CAMP factor and hyaluronate lyase, with largely increased abundance in the Δ*prsA1* mutant which exhibits extremely high SpeB activity (Figure 3(a) and Table S2). In addition, we found that Δ*prsA1*, Δ*prsA2*, and Δ*prsA1/A2* mutants remained defective in biofilm formation even in the presence of SpeB inhibitor E-64 which inhibited SpeB maturation (Figure 4(a)). This observation indicates that PrsA1 itself is sufficient to affect some GAS surface proteins and contributes to the related phenotype in spite of excessive production of mature SpeB. This finding suggests that PrsA clearly has its unique role in shaping the GAS proteome in a SpeB-independent manner.

In summary, *prsA* inactivation in GAS resulted in gross proteomic changes, especially those involved in metabolism, protein transport and virulence determinants. Although the detailed molecular mechanism attributed to PrsA’s action is not fully understood, the importance of PrsA in regulating multifaceted bacterial traits is clear. Further investigation to delineate the relationship between PrsA1 and PrsA2 and identification of PrsA’s substrates may facilitate the development of therapeutic strategies to limit the progression of the infection and ameliorate complications associated with GAS infection.

## Materials and methods

### Bacterial strains and cell culture

GAS clinical isolates A20 (*emm*1) and 4063-05 (*emm*4) used in this study have been previously described [22,38]. GAS strains were cultured in static liquid Todd-Hewitt broth (THB, Acumedia) containing 2% yeast extract (Acumedia). Bacteria were grown to mid-log phase for experiments except where indicated. HaCaT, a human keratinocyte cell line [39], was maintained in DMEM supplemented with 10% FBS.

### Construction of ΔprsA strains and prsA complementation

A precise, in-frame allelic replacement of the *prsA1* gene or *prsA2* gene with the chloramphenicol acetyltransferase (*cat*) gene was generated in M1 (strain A20) and M4 (strain 4063-05) GAS, respectively, using a previously described method [40] with primers listed in Table S1. Briefly, DNA fragments (~900 bp) immediately upstream and downstream of *prsA1 and prsA2* were individually PCR amplified from chromosomal DNA using primers with 19-bp extensions matching the 5ʹ and 3ʹ end of the *cat* gene. The flanking sequence PCR products were then joined with the *cat* gene by fusion PCR, and the resulting amplicon was cloned into temperature-sensitive suicide erythromycin-resistant vector pHY304 to generate the knockout vector, pHY-prsA1 and pHY-prsA2. This vector was transformed into GAS by electroporation, and single recombination events were identified at 37°C under 5 µg/ml erythromycin selection. Selection was relaxed by serial passage at 30°C without antibiotics, and GAS experienced double-crossover events was selected for the loss of erythromycin resistance. The replacement of the target gene by *cat* was verified by PCR using appropriate primers listed in Table S1. For *prsA* complementation, full-length *prsA1 and prsA2* genes were amplified by primers listed in Table S1, and PCR products were cloned into the pLZ12Km2-P23R-TA plasmid (gift from Dr Thomas Proft, University of Auckland) carrying the kanamycin-resistant gene, to create pLZ-prsA1 and pLZ-prsA2. GAS strains used in this study are listed in Table 1.

### Generation of recombinant His-tagged PrsA proteins and anti-PrsA antibodies

To produce the N-terminal His-tagged PrsA1 and PrsA2, the GAS chromosomal DNA was used for gene amplification of the *prsA1* and *prsA2* genes without the predicted signal-peptide coding sequences with primers listed in Table S1. The amplified ORFs were cloned into the pET15b vector (Novagen) using XhoI and BamHI restriction sites and expressed in *E. coli* BL21 (ED3) by IPTG induction for 16 h at 30°C. The recombinant proteins were purified using Ni-NTA beads (Qiagen) according to manufacturer’s instructions. The rabbit anti‐PrsA1 and anti-PrsA2 polyclonal antibodies were generated by LTK Biolaboratories Company (Taoyuan, Taiwan) against the above-mentioned recombinant His-tagged PrsA1 and PrsA2 proteins.

### Growth curve

To determine the growth rates of GAS strains, overnight cultures of WT, Δ*prsA1*, Δ*prsA2* and Δ*prsA1/A2* were diluted with fresh medium to optical densities at wavelength 600 nm (OD_600_) of 0.1 and incubated for 5 hours at 37°C. Cultures were vortexed at each time point and the value of OD_600_ was measured with the use of SPECTRONIC™ 200 Spectrophotometer (Thermo Fisher Scientific).

### Scanning electron microscopic (SEM) analysis

SEM analysis was performed as previously described [41,42]. Briefly, GAS was fixed with 2% glutaraldehyde-RPMI 1640 for 1 h at room temperature, washed with distilled water, dehydrated with 100% *t*-butyl alcohol and freeze-dried. Samples were coated with platinum and examined using an emission-SEM (JSM-6390LVZ with SEM control user interface software version 8.16; JEOL Ltd., Japan).

### Transmission electron microscopy (TEM) analysis

GAS was prefixed with 2.5% glutaraldehyde. The specimens were collected by centrifugation and washed once in 0.15 M phosphate buffer. The supernatant was removed and warm 2% agar was added. After cooling, the embedded pellet was cut into pieces. After washing three times, the pieces were postfixed in 1% osmium tetroxide for 6 h. The osmium tetroxide was removed through repeated washings three times with 0.15 M phosphate buffer (15 min) and dehydrated in graded series of ethanol and embedded in epoxy resin (Quetol 812, Nisshin EM, Tokyo). Ultrathin sections were made with a diamond knife on an ultramicrotome (Leica UC7), stained in 2% uranyl acetate (20 min) and 0.4% lead citrate (5 min), and observed under a Hitachi H-7500 electron microscope at an accelerating voltage of 80 kV.

### Preparations of bacterial secreted proteins, extracellular vesicles, and crude membrane extracts

For secreted proteins, overnight culture supernatants were passed through 0.22 µm filter to remove potential bacterial debris, followed by 10% trichloroacetic acid (TCA) precipitation to recover the total secreted proteins. To harvest bacterial extracellular vesicles, filtered overnight culture supernatants were centrifuged at 150,000 g for 3 h at 4°C. For membrane protein preparation, stationary phase GAS was resuspended in KPN buffer (20 mM potassium phosphate, 140 mM NaCl [pH 7.5]) containing lysozyme (Sigma, 400 µg/ml), RNase (Sigma, 6 µg/ml), and DNase (Sigma, 6 µg/ml) and protease cocktail inhibitors (Roche), incubated at room temperature for 10 min, and sonicated with Vibra-Cell™ VX130 (Sonics & Materials) to completely disrupt bacterial cells. The bacterial lysates were first centrifuged at 10,000 g to remove cell debris, and the supernatants were collected and further centrifuged at 120,000 g to precipitate the crude membrane fraction. The collected proteins were separated on 10% SDS-PAGE and visualized by silver staining (Bio-Rad) or subjected to quantitative mass spectrometry analysis.

### TMT labeling and mass spectrometry

Samples were immersed in equal volumes of 8 M urea with 50 mM HEPES (pH = 8.5) and a lysis buffer containing 75 mM NaCl (Sigma), 3% sodium dodecyl sulfate (SDS; Fisher), 1 mM NaF (Sigma), 1 mM beta-glycerophosphate (Sigma), 1 mM sodium orthovanadate (Sigma), 10 mM sodium pyrophosphate (Sigma), 1 mM phenylmethylsulfonyl fluoride (Sigma), 1× cOmplete Mini EDTA-free protease inhibitors (Roche), and 50 mM HEPES (Sigma) (pH 8.5). Samples were subjected to probe sonication. Denatured proteins were next subjected to reducing conditions in 5 mM dithiothreitol (DTT), alkylation in 15 mM iodoacetamide (IAA), and quenching in 5 mM DTT. Proteins were precipitated via addition of TCA to the solution. Samples were washed in ice-cold acetone three times and dried on a heating block. Pellets were resuspended in a solution of 1 M urea with 50 mM HEPES (pH = 8.5) and subjected to enzymatic digestion with LysC and sequencing-grade trypsin overnight at room temperature and for 6 h at 37°C, respectively. Digested peptides were desalted on C18 columns (Waters) using protocols recommended by the manufacturer. Peptides were dried under vacuum and quantified using a commercially available pepquant kit (Pierce). 50 µg aliquots were separated and dried under vacuum for tandem mass tag labeling. Dried aliquots were resuspended in 50% acetonitrile and 200 mM HEPES (pH = 8.5). TMT labels were added to the samples and allowed to incubate for 1 h at room temperature. The reaction was quenched via the addition of a 5% solution of hydroxylamine and incubation at room temperature for 15 min. Samples were mixed and desalted on C18 columns. Desalted multiplexed samples were dried under vacuum. Multiplexed samples were next fractionated in an Ultimate 3000 high-performance liquid chromatography (HPLC) system with a fraction collector, degasser, and variable-wavelength detector. Separation was performed using a C18 column (Thermo Scientific) (4.6 mm by 250 mm) on a 22% to 35% 60 min gradient of acetonitrile and 10 mM ammonium bicarbonate (ABC) (Fisher) at 0.5 ml/min. The resulting 96 fractions were combined as previously described [43]. Fractions were dried under vacuum. Fractions were next analyzed using tandem mass spectrometry (MS2/MS3) on an Orbitrap Fusion mass spectrometer (Thermo Fisher Scientific) with an in-line EASY-nLC 1000 instrument (Thermo Fisher Scientific). Separation and acquisition settings were performed using the previously defined methods [44].

### Proteome data analysis and annotation

Mass spectrometry data was subjected to database search using Proteome Discoverer 2.1 software. Data was searched against the reference proteome for GAS MGAS10750 M4 serotype downloaded from Uniprot.com on 1/30/2019. The SEQUEST search algorithm was employed to align MS2 spectral data against theoretical peptides generated *in silico* [45]. Precursor tolerance was set to 50 ppm and fragment tolerance was set to 0.6 Da. Static modifications were specified for TMT labels on N-termini and lysine residues, as well as for carbamidomethylation of cysteines. Dynamic modifications were set for oxidation of methionine. A 1% false discovery rate was specified for the decoy database search [46]. Peptide spectral match abundances were summed to the protein level and resultant summed values were normalized against the average value for each protein divided by the median of all average protein values. A second normalization step was performed whereby the abundance value for each protein per sample was divided by the median value for each channel which had itself been divided by the overall dataset median. Statistical analyses were performed in Excel. Differentially abundant proteins were identified using π score, a significance metric that incorporates both fold changes and traditional *p*-value based significance scores, determined through a Student’s *t* test with or without Welch’s correction [47]. Figures based on proteome data were generated in GraphPad Prism and in BioVenn [48].

### Data and code availability

Proteome data was uploaded to massive.ucsd.edu and the ProteomeXchange under the identifier PXD020477 for secreted proteins in the culture supernatants and PXD020476 for extracellular vesicles.

### Western blot analysis

Bacterial culture supernatants were collected from stationary phase GAS, passed through 0.22 µm filter and precipitated by 10% TCA. The recovered proteins were separated in an SDS-PAGE gel and transferred to the PVDF membrane (Invitrogen), probed with antibodies recognizing SpeB (Abcam), T4 antigen (Abcam), SLO (GeneTex), PrsA1, and PrsA2, and visualized with a Li-Cor Odyssey scanner after addition of IRDye® 800CW-conjugated secondary antibodies.

### SpeB activity assay

SpeB activity assays were performed as previously described [49] with slight modification. Filtered supernatant (200 µl) collected from stationary phase GAS was mixed with 200 µl of activation buffer (1 mM EDTA, 20 mM DTT in 0.1 M sodium acetate buffer, pH 5.0) and incubated for 30 min at 40°C. After activation, 400 µl of 2% (w/v) azocasein in activation buffer was added and incubated for 1 h at 40°C. TCA was then added to a final concentration of 2% (w/v) and thoroughly mixed. The mixture was then centrifuged at 15,000 g for 5 min and the OD_440_ value of the resulting supernatants was determined.

## Biofilm assay

Log phase GAS were adjusted to 10^5^ CFU/ml with C medium (0.5% Proteose Peptone no.3, 1.5% yeast extract, 10 mM K_2_HPO_4_, 0.4 mM MgSO_4_, 17 mM NaCl), seeded into a 96-well plate and incubated at 37°C for 24 h in the presence or absence of SpeB inhibitor E-64 (Sigma). After removal of medium, the plates were washed three times with PBS, fixed with methanol, and stained with 0.2% crystal violet at room temperature for 10 min. After extensive PBS washes, the bound dye was extracted with 100 µl of 1% SDS and quantitated by measuring the solution absorbance at 540 nm.

### Assays for bacterial adherence and infection-induced cytotoxicity

HaCaT cells (2 × 10^5^ cells/well) were plated on a 24-well plate 1 day prior to the assay, infected with 5 × 10^6^ CFU GAS per well, and centrifuged for 5 min at 1600 rpm to initiate bacterial contact. After 30 min of incubation and extensive washes with PBS, infected cells were detached with 5 mM EDTA/PBS and disrupted using 0.025% Triton X-100. Surviving bacterial CFUs were quantified by serial dilution plating on THY plates. To measure GAS-induced cell death, HaCaT cells (2.5 × 10^4^ cells/well) were plated on a 96-well plate 1 day prior to the experiment and infected with GAS at MOI of 3 and 10 for 1 h, extensively washed, and added penicillin and gentamicin to 10 and 100 µg/ml, respectively. After 24 h infection, cells were washed, fixed with methanol and incubated with 0.2% crystal violet at room temperature for 10 min to stain viable cells. Stained cells were extensively washed with PBS and the bound dye was recovered with 100 µl of 1% SDS and measured the solution absorbance at 540 nm.

### Mouse soft tissue infection model

All mouse experiments were conducted under a protocol approved by the National Taiwan University College of Medicine Animal Care and Use Committee (IACUC 20180463). Female ICR mice (6-weeks old, n = 10) were subcutaneously infected with WT and *prsA* mutants (10^8^ CFU/mouse) in the shaved right and left back flank, respectively. Mice were anaesthetized by 2% isoflurane and lesion sizes were recorded by camera with a fixed height and calculated by Image J software.

### Statistical analysis

All statistical tests were performed using GraphPad Prism version 8 (GraphPad Software, Inc.). The data presented here were combined from 2 to 3 independent experiments and expressed as mean ± SD except where indicated. A two-tailed *t* test or one-way ANOVA with Tukey’s multiple comparison posttest was used to compare the data were indicated in the legends. A *p*-value <0.05 was considered significant for all tests.

## Supplementary Material

Supplemental Material
