# Supplementary material for: Unique virulence role of post-translocational chaperone PrsA in shaping Streptococcus pyogenes secretome
Source: Virulence. 2021 Oct 1;12(1):2633–47. doi: 10.1080/21505594.2021.1982501 (PMC8489961; doi:10.1080/21505594.2021.1982501)
Supplement: Supplemental Material [file KVIR_A_1982501_SM3960.zip › supplementary/Revised2_Table S1.docx]

**Supplementary Table 1**

| **Primers** | **Description** |
| --- | --- |
| **Primers used for constructing knockout plasmid** |  |
| cgactcgagGGATCGAAATCTTCACCACG cagtgatttttttctccatGTAGGATTTTATTGACTAATCAG | Upstream flanking region of *prsA1* |
| gtggcagggcggggcgtaaTTACTTCTGAGCTCCTTTTTAT cgaggatccGGACTCATAATTATCTTCTCAC | Downstream flanking region of *prsA1* |
| cgactcgagATGGGTTGCTCCTTTGCTGG cagtgatttttttctccatagggttgccagtttatttccc | Upstream flanking region of M1 *prsA2* |
| gtggcagggcggggcgtaaTAATAATTACTGTTATCTTATATC cgaggatccaacagaacctgaggcacg | Downstream flanking region of M1 *prsA2* |
| cgactcgagcgctagcggtactagtattag CAGTGATTTTTTTCTCCATtgccagtttatttccctaaggt | Upstream flanking region of M4 *prsA2* |
| Gtggcagggcggggcgtaataataattactgttatcttata cgaggatccgtgtcggtaaagtaggcg | Downstream flanking region of M4 *prsA2* |
|  |  |
| **Primers used for *prsA* knockout validation** |  |
| ATGGAGAAAAAAATCACTGGATATACC TTACGCCCCGCCCTGCCACTCATCGCA | *cat* amplification |
| GAACGTGGTACGGCTGGG TGACAGCTTGGGCAATAGC | *prsA1* Up-Dn amplification |
| GTCCTGTCATCGTAGTATCT ccaaccagtatactaccaag | M1 *prsA2* Up-Dn amplification |
| Gtagtggaagcagatgctgc gtagacatggattatggtcca  TTATTGTGCAGCTGGCTCTT ATGAAAAACTCAAATAAACTCATT  cagctgcagATGAGAAAAGAGGCTCAAAAG cagagatctCTATTTTTGGGCAGGTTGTTT | M4 *prsA2* Up-Dn amplification  *prsA1* amplification  *prsA2* amplification |
| **Primers used to generate *prsA* complementation plasmid** |  |
| gcggccgcAAATATATTGTCTTTCTACCAGAGAT tctagaTTATTGTGCAGCTGGCTCTT | *prsA1* complementation |
| gcggccgcGTTTCACGCCGGCCTAAGCA tctagaCTATTTTTGGGCAGGTTGTTT | *prsA2* complementation |
| **Primers used to generate recombinant His-tagged PrsA**  ctcgagTGTCAATCAACTAATGACAATAC  ggatccTTATTGTGCAGCTGGCTCTTG  ctcgagTGTCAATCATCACACAACAACA  ggatccCTATTTTTGGGCAGGTTGTTTT | Generation of His-PrsA1  Generation of His-PrsA2 |
