# Supplementary material for: Unique virulence role of post-translocational chaperone PrsA in shaping Streptococcus pyogenes secretome
Source: Virulence. 2021 Oct 1;12(1):2633–47. doi: 10.1080/21505594.2021.1982501 (PMC8489961; doi:10.1080/21505594.2021.1982501)
Supplement: Supplemental Material [file KVIR_A_1982501_SM3960.zip › supplementary/Revised2_table S4-S7.docx]

**Table S4. Exoproteins with significantly increased abundance in *prsA* deficient mutants**

| Δ*PrsA1* only (47) | AroE, ArtQ, AtpF, CitE, ClpP, CopY, DnaA, FolC1, GlpO, GlyS, HylA, LacA, LigA, MetN, NanH, PepD, Ppc, RlmN, RpmE2, RpmJ, ScrR, ThiD, TlpA, VicR, XerS, Spy0033, Spy0040, Spy0220, Spy0291, Spy0477, Spy0508, Spy0511, Spy0678, Spy0794, Spy0986, Spy1081, Spy1123, Spy1198, Spy1211, Spy1327, Spy1383, Spy1422, Spy1440, Spy1444, Spy1514, Spy1803, Spy1880 |
| --- | --- |
| Δ*PrsA2* only (39) | ArgS, AroB, AspS, DnaD, Dyr, FabK, FolD, MurI, MvaK2, ParB, PlsX, PolA, ProA, ProC, PurB, RgpDc, RgpEc, RlmH, Rnc, RnhB, RplT, RpsE, RpsG, RpsJ, RpsO, RsmA, TsaD, Spy0127, Spy0292, Spy0386, Spy0553, Spy0557, Spy0656, Spy0735, Spy1027, Spy1031, Spy1061, Spy1120, Spy1329 |
| Δ*PrsA1/A2* only (60) | AdcC, AmyA, ClpC, ClpE, ClpL, CpsFQ, CysS, DacA1, CshA, FruR, GatB, Isp, LysS, LytR, Mur1-2, OppB, Pbp1b, Pbp2A, PolC, PrgA, PstS, PyrD, RecD, RplC, RplD, RplS, RplW, RpsC, SecA, SecY, SibA, Spi, Thy, Xpt, Spy0010, Spy0039, Spy0380, Spy0575, Spy0576, Spy0579, Spy0587, Spy0622, Spy0710, Spy0717, Spy0734, Spy0895, Spy1108, Spy1116, Spy1194, Spy1303, Spy1322, Spy1331, Spy1373, Spy1409, Spy1476, Spy1664, Spy1707, Spy1820, Spy1946, Spy1975 |
| Δ*PrsA1* & Δ*PrsA2 (17)* | AhrC2, DnaB, HflX, MutM, OppD, PflC, ProB, RecJ, RpsB, Tmk, XseA, Spy0271, Spy0516, Spy1142, Spy1555, Spy1827, Spy1953 |
| Δ*PrsA1* & Δ*PrsA1/A2 (38)* | AckA, AsnS, CovS, CshB, DeaD2, FtsE, Gmk, GyrA, InfB, Isp2, LacR2, ManM, ManN, MutS2, NadK, OpuABC, PacL, Pbp1A, PstB1, PurR, RecX, RofA, SipC, TopA, Spy0041, Spy0233, Spy0415, Spy0696, Spy0937, Spy1132, Spy1333, Spy1355, Spy1379, Spy1502, Spy1804, Spy1851, Spy1852, Spy1947 |
| Δ*PrsA2* & Δ*PrsA1/A2 (20)* | EcfA2, Era, FabH, Ffh, Fhs1, FolP, GyrB, HisS, MetS, NadE, NifS1, NifS2, RecA, RnhC, UvrA, Spy0815, Spy1115, Spy1460, Spy1574, Spy1651 |
| Δ*PrsA1*, Δ*PrsA2* & Δ*PrsA1/A2 (33)* | AgaS, AroA, AsnA, BirA, CarB, Cfa, CinA, DltD, EngB, FolE, FtsA, GlnQ2, GlyA, LipL, MetB, MnmE, MscL, MurZ, NrdD, OpuAA, PheT, QueA, Rex, Rgg, RpsD, RpsL, SpeB, Spy0255, Spy0502, Spy0737, Spy0785, Spy1278, Spy1471 |

**Table S5. exoproteins with significantly decreased abundance in *prsA* deficient mutants**

| Δ*PrsA1* only (31) | BcaT, GlmM, HsdM, InfC, NusB, PyrH, RimM, RplA, RplB, RplE, RplM, RplQ, RplR, RpmA, RpmI, RpoD, RpsZ, TruA, Spy0046, Spy0204, Spy0522, Spy0676, Spy0681, Spy0809, Spy0994, Spy1322, Spy1332, Spy1346, Spy1493, Spy1655, Spy1965 |
| --- | --- |
| Δ*PrsA2* only (51) | AcoC, DppA, Emm, EndoS, GlnH, GreA, LppC, MapZ, PepB, Pnp, PulA, RmlD, Shp, SmeZ, SpeJ, Spy0040, Spy0114, Spy0116, Spy0142, Spy0149, Spy0196, Spy0207, Spy0231, Spy0265, Spy0266, Spy0365, Spy0368, Spy0373, Spy0383, Spy0499, Spy0519, Spy0603, Spy0609, Spy0653, Spy0678, Spy0709, Spy0742, Spy0762, Spy0928, Spy0969, Spy1024, Spy1091, Spy1157, Spy1220, Spy1330, Spy1589, Spy1747, Spy1810, Spy1819, Spy1921, Spy1964 |
| Δ*PrsA1/A2* only (50) | ApbA, ArgR, Cbf, CoaD, ComEB, Ddl, Def, DnaN, Dtd, FabZ, Frr, GlgP, Hyl, Irr, MipB, Nox, PepC, PepF, PepN, PepT, Pfl, Pfs, Pgi, PgmA, Plr, PpaC, Prs, PrsA2^#^, RecO, Rnj, RnpA, Rnz, RplL, RpsP, SmpB, Tal, UviB, Spy0108, Spy0256, Spy0304, Spy0435, Spy0674, Spy0998, Spy1025, Spy1121, Spy1213, Spy1342, Spy1385, Spy1523, Spy1966 |
| Δ*PrsA1* & Δ*PrsA2 (3)* | NifS3, Snf, Spy1113 |
| Δ*PrsA1* & Δ*PrsA1/A2 (19)* | AcoL, AcpP, AdhA, CpsY, FolK, GroS, Idh, MalX, PyrC, RplN, RplU, RpsH, SalR, Upp, Spy0447, Spy0713, Spy0763, Spy0940, Spy1753 |
| Δ*PrsA2* & Δ*PrsA1/A2 (43)* | AhpC, ArcA, ArcB, ArcC, AtpD, CitF, CpsFP, CutC, DeoD, DnaK, Eno, GabD, GapN, GlnA, Gor, GroL, Hpf, Ifs, LacB, Mac, MutT, NrdF2, PfkA, PppL, PrsA2, RimP, Spd, Spd3, SpeA2, Ssb2, PrsA1, Tkt, TpiA, Spy0115, Spy0118, Spy0240, Spy0291, Spy0357, Spy0512, Spy0630, Spy1562, Spy1587, Spy1822 |
| Δ*PrsA1*, Δ*PrsA2* & Δ*PrsA1/A2 (49)* | Adk, AtpA, DeoB, DltA, EstA, Fba, FruA, GlcA, GloA, GuaA, Map, MsrA2, Nga, NusG, PepO, PerR, Pgk, PtsB, PtsH, RpoY, RpsF, SclA, ScpA, Slo, SpyCEP, TrmFO, Tsf, Udp, Spy0117, Spy0148, Spy0264, Spy0339, Spy0354, Spy0355, Spy0448, Spy0808, Spy0914, Spy0941, Spy1080, Spy1192, Spy1395, Spy1396, Spy1469, Spy1630, Spy1692, Spy1806, Spy1817, Spy1823, Spy1888 |

^#^ribose-phosphate pyrophosphokinase

**Table S6. EV proteins with significantly increased abundance in *prsA* deficient mutants**

| Δ*PrsA1* only (36) | AmyA, AmyB, ArtQ, Cfa, FtsA, GlpK, LacG, Mur1-1, MutR, NrdF1, PhoH, PstB2, PulA, RofA, SalB, SpeJ, SrtT, SunL, YajC, Spy0010, Spy0099, Spy0271, Spy0579, Spy0584, Spy0592, Spy0737, Spy0958, Spy1115, Spy1146, Spy1157, Spy1211, Spy1333, Spy1346, Spy1536, Spy1764, Spy1871 |
| --- | --- |
| Δ*PrsA2* only (33) | Apt, AroD, CinA, Def, DngA, DnaJ, Gmk, PurC, PurK, RlmN, RnhB, Rnr, RplK, RplL, SpeB, XseA, Spy0038, Spy0087, Spy0110, Spy0197, Spy0361, Spy0492, Spy0534, Spy0552, Spy0574, Spy0587, Spy0635, Spy1065, Spy1107, Spy1195, Spy1372, Spy1827, Spy1842 |
| Δ*PrsA1/A2* only (110) | AcoA, AcoB, AlaS, AroA, AroC, ClpE, ClpL, ClpX, CpsFO, CpsFQ, CshA, CshB, DeaD2, DnaG, DpfB, EngB, Era, ExoA, FabD, FabF, Fmt, FolP, FtsZ, FusA, GatA, GpsA, GuaB, HisS, HolB, HsdM, HsdS, HslO InfB, LacR1, LepA, MetK, MnmE, MurC, MurD, MurF, NagA, NrdR, OadA2, Obg, ParB, ParE, PcrA, PrfA, PrfB, PriA, PurA, PyrC, PyrH, QueA, RecJ, RnmV, RqcH, RplA, RplB, RplC, RplF, RplJ, RplO, RplU, RplV, RplW, RpsC, RsmH, SibA, ThiI, TrmD, TrpS, TruB, Tuf, Udk, VicR, Xpt, YchF, Spy0029, Spy0041, Spy0046, Spy0161, Spy0220, Spy0255, Spy0298, Spy0332, Spy0464, Spy0493, Spy0553, Spy0593, Spy0627, Spy0670, Spy0734, Spy0765, Spy0831, Spy1014, Spy1017, Spy1030, Spy1082, Spy1106, Spy1212, Spy1245, Spy1370, Spy1386, Spy1440, Spy1651, Spy1660, Spy1746, Spy1785, Spy1915, |
| Δ*PrsA1* & Δ*PrsA2 (14)* | Fms, MccF, Rgg, Spy0034, Spy0191, Spy0192, Spy0239, Spy0390, Spy0511, Spy0573, Spy0585, Spy0853, Spy0865, Spy1329 |
| Δ*PrsA1* & Δ*PrsA1/A2 (66)* | AccC, AckA, AmiC, AsnS, AspC, ClpC, DexS, DnaA, DnaB, DnaE, DnaX, GlmS, GyrA, GyrB, HemN, HrcA, HsdR, InlA, Isp, Isp2, LacD, LacR2, MetK1, Mur1-2, MurA, MutL, MutS, MutS2, NadK, NrdD, NrdE1, ParC, PepX, PflD, PstB1, PyrB, RadA, RecG, RelA, RuvB, Smc, Snf, ThiD, ThyA, TrcF, UvrA, UvrB, UvrC, Spy0453, Spy0517, Spy0710, Spy0892, Spy1003, Spy1070, Spy1078, Spy1176, Spy1194, Spy1203, Spy1253, Spy1406, Spy1414, Spy1428, Spy1460, Spy1537, Spy1573, Spy1880 |
| Δ*PrsA2* & Δ*PrsA1/A2 (40)* | AsnA, BcaT, CarA, DnaI, FolE, FruR, GatB, GltX, GlyA, GpmA, GuaC, MecA, MetB, MetS, MvaD, MvaS1, NadD, NifS2, NrdE2, PheT, PrmA, ProB, Pyk, QueH, RecD, RplD, Tgt, TrmFO, TrpG, Spy0261, Spy0380, Spy0386, Spy0445, Spy0531, Spy0818, Spy0827, Spy0919, Spy0992, Spy1843, Spy1974 |
| Δ*PrsA1*, Δ*PrsA2* & Δ*PrsA1/A2 (37)* | AgaS, AhrC2, ArgS, AspS, BirA, CarB, Cca, CitC, CovS, CrgR, Fps, GlyS, HflX, LigA, LysS, MurE, MurZ, NadE, OpuAA, PolC, Ppc, PrfC, RecN, SerS, TopA, BipA, VicX, Spy0331, Spy0502, Spy0515, Spy0598, Spy0902, Spy1063, Spy1335, Spy1549, Spy1789, Spy1899 |

**Table S7. EV proteins with significantly decreased abundance in *prsA* deficient mutants**

| Δ*PrsA1* only (24) | AhpC, Cas2, CitX, CutC, DeoB, GlmU, GloA, MapZ, MvaK1, NanH, Rnz, RsfS, SatD, Upp, Spy0100, Spy0106, Spy0173, Spy0373, Spy0674, Spy1397, Spy1504, Spy1514, Spy1552, Spy1744 |
| --- | --- |
| Δ*PrsA2* only (15) | DacA2, DppA, EndoS, FtsH, MurM, NtpE, Slo, SunL, Spy0149, Spy339, Spy0653, Spy1090, Spy1395, Spy1692, Spy1791 |
| Δ*PrsA1/A2* only (129) | AgaD, Alr, ArcA, ArcB, AroE2, ArtQ, AtpE, Cbf, CdsA, CitE, CitF, CoaA, CopA, CpsX, DeoD, Dfp, Dpr, FabZ, FlaR, FolK, GabD, GapN, GldA, GlnA, Gor, GroL, GroS, HutH, HutI, Hyl, MalA, MalC, MalD, ManM, ManN, MefE, MurG, NtpI, NtpK, NupC, OppB, OppC, Pbp1A, PepA, PepB, PepC, PepD, PepF, PepN, PepO, PfkA, Pgi, Pnp, PotB, PppL, ProB, Prs, PtsD, PtsH, PtsK, PunA, RgpAc, RgpBc, Rpe, SagC, ScrA, SecY, SodA, SptS, SrtK, VicK, YvqE, Spy0043, Spy0139, Spy0142, Spy0233, Spy0268, Spy0272, Spy0333, Spy0384, Spy0403, Spy0438, Spy0439, Spy0499, Spy0552, Spy0564, Spy0601, Spy0603, Spy0624, Spy0678, Spy0700, Spy0798, Spy0820, Spy0826, Spy0891, Spy0936, Spy0969, Spy0980, Spy0990, Spy1049, Spy1053, Spy1081, Spy1088, Spy1091, Spy1130, Spy1143, Spy1162, Spy1193, Spy1213, Spy1288, Spy1342, Spy1355, Spy1379, Spy1396, Spy1408, Spy1468, Spy1469, Spy1498, Spy1562, Spy1583, Spy1584, Spy1587, Spy1608, Spy1659, Spy1823, Spy1883, Spy1886, Spy1945, Spy1969 |
| Δ*PrsA1* & Δ*PrsA2 (1)* | RplP |
| Δ*PrsA1* & Δ*PrsA1/A2 (28)* | ArcC, AtpG, CitD, Def, DppC, Emm, Eno, FruA, GlcK, IdnO, NusG, Pfs, Plr, PolA, PpaC, Pta, RpoY, Tal, Tkt, Spy0383, Spy0561, Spy0878, Spy0914, Spy0941, Spy1089, Spy1120, Spy1378, Spy1964 |
| Δ*PrsA2* & Δ*PrsA1/A2 (19)* | CzcD, HutU, HylA, Ifs, LacE, Mac, PrsA2, ScpA, SpeA2, YidC, Spy0114, Spy0115, Spy0116, Spy0117, Spy0121, Spy0742, Spy1125, Spy1512, Spy1822 |
| Δ*PrsA1*, Δ*PrsA2* & Δ*PrsA1/A2 (16)* | Cas1, DivIB, DppD, DppE, GlgP, Nga, PtsB, PtsC, SagG, SagH, SagI, Spd3, SpyCEP, SrtF, PrsA1, Spy0118 |
