# Supplementary material for: Unique virulence role of post-translocational chaperone PrsA in shaping Streptococcus pyogenes secretome
Source: Virulence. 2021 Oct 1;12(1):2633–47. doi: 10.1080/21505594.2021.1982501 (PMC8489961; doi:10.1080/21505594.2021.1982501)
Supplement: Supplemental Material [file KVIR_A_1982501_SM3960.zip › supplementary/Supplementary Legends.docx]

**Supplementary Table Legends**

**Supplementary Table 1**. Primers used in this study.

**Supplementary Table 2.** Normalized abundance of exoproteins detected from WT and *prsA* single and double deletion mutants by tandem mas tag proteomics.

**Supplementary Table 3.** Normalized abundance of extracellular vesicle (EV) proteins detected from WT and *prsA* single and double deletion mutants by tandem mas tag proteomics.

**Supplementary Table 4.** Exoproteins with significantly increased abundance in *prsA* deficient mutants.

**Supplementary Table 5.** Exoproteins with significantly decreased abundance in *prsA* deficient mutants.

**Supplementary Table 6.** EV proteins with significantly increased abundance in *prsA* deficient mutants.

**Supplementary Table 7.** EV proteins with significantly decreased abundance in *prsA* deficient mutants.

**Supplementary Figure Legends**

**Supplementary Figure 1.** (A) Schematic representation of genes neighboring *prsA1* and *prsA2*. *spy1238*, Cro/CI family transcriptional regulator; *alaS*, alanyl-tRNA synthetase; *prsA1*, peptidyl-prolyl cis-trans isomerase; *spy1241*, O-methyltransferase; *spy1242*, permease MFS superfamily; *pepB*, oligoendopeptidase F; *spy1823*, fibronectin-binding protein; *spy1824*, hypothetical cytosolic protein; *prsA2*, peptidyl-prolyl cis-trans isomerase; *spy1826*, hypothetical protein; *spy1827*, spi family protease inhibitor; *speB*, streptococcal cysteine protease. (B) Verification of the *prsA* knockout in GAS. Chromosomal DNA was extracted from WT and mutants and five pairs of primer were used to demonstrate the deletion of *prsA1* and *prsA2*. Morphology of M4 WT and its *prsA* deletion derivatives were examined by SEM (C) and TEM (D)

**Supplementary Figure 2.** Spearman correlation of TMT proteome replicates in exoproteome (A) and EV proteome (B). (C) Overview of protein domains of GAS PrsA1 and PrsA2.

**Supplementary Figure 3. Examination of the exoprotein profile and the maturation and proteolytic activity of SpeB in M1 GAS and its isogenic *prsA* deletion mutants.** (A) SDS-PAGE and silver stain analysis of proteins collected from cell-free culture media. (B) Expression of SpeB in the cell-free culture supernatant by Western blot analysis. (C) Expression of PrsA in the *prsA*-complemented M1 GAS strains. (D) Expression and maturation of SpeB in the *prsA*-complemented M1 GAS strains. (E) SpeB-mediated proteolytic activities.
