# Supplementary figures and images for: Unique virulence role of post-translocational chaperone PrsA in shaping Streptococcus pyogenes secretome
Source: Virulence. 2021 Oct 1;12(1):2633–47. doi: 10.1080/21505594.2021.1982501 (PMC8489961; doi:10.1080/21505594.2021.1982501)

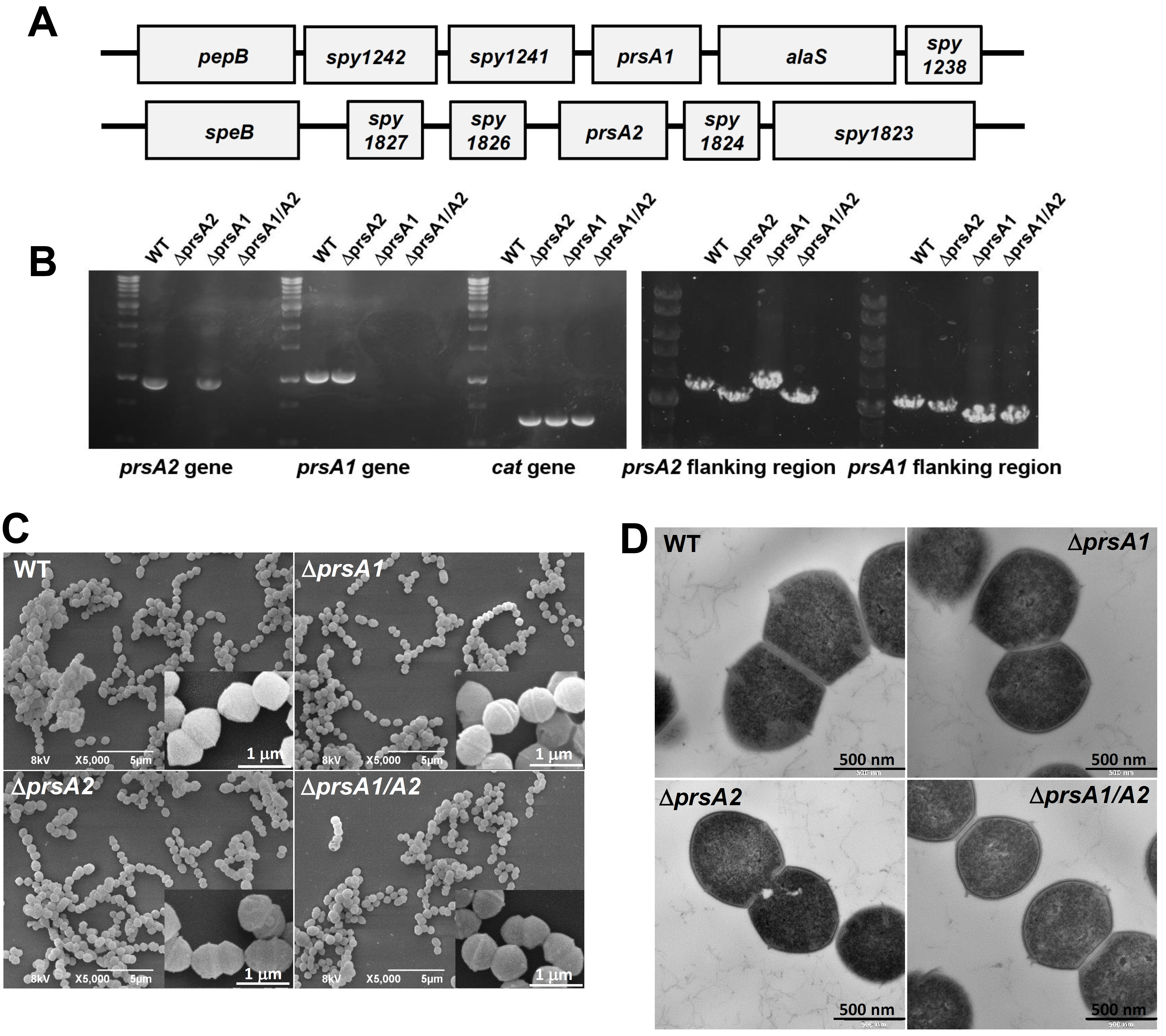

Supplement: Supplemental Material [file KVIR_A_1982501_SM3960.zip › supplementary/Fig_S1.jpg]

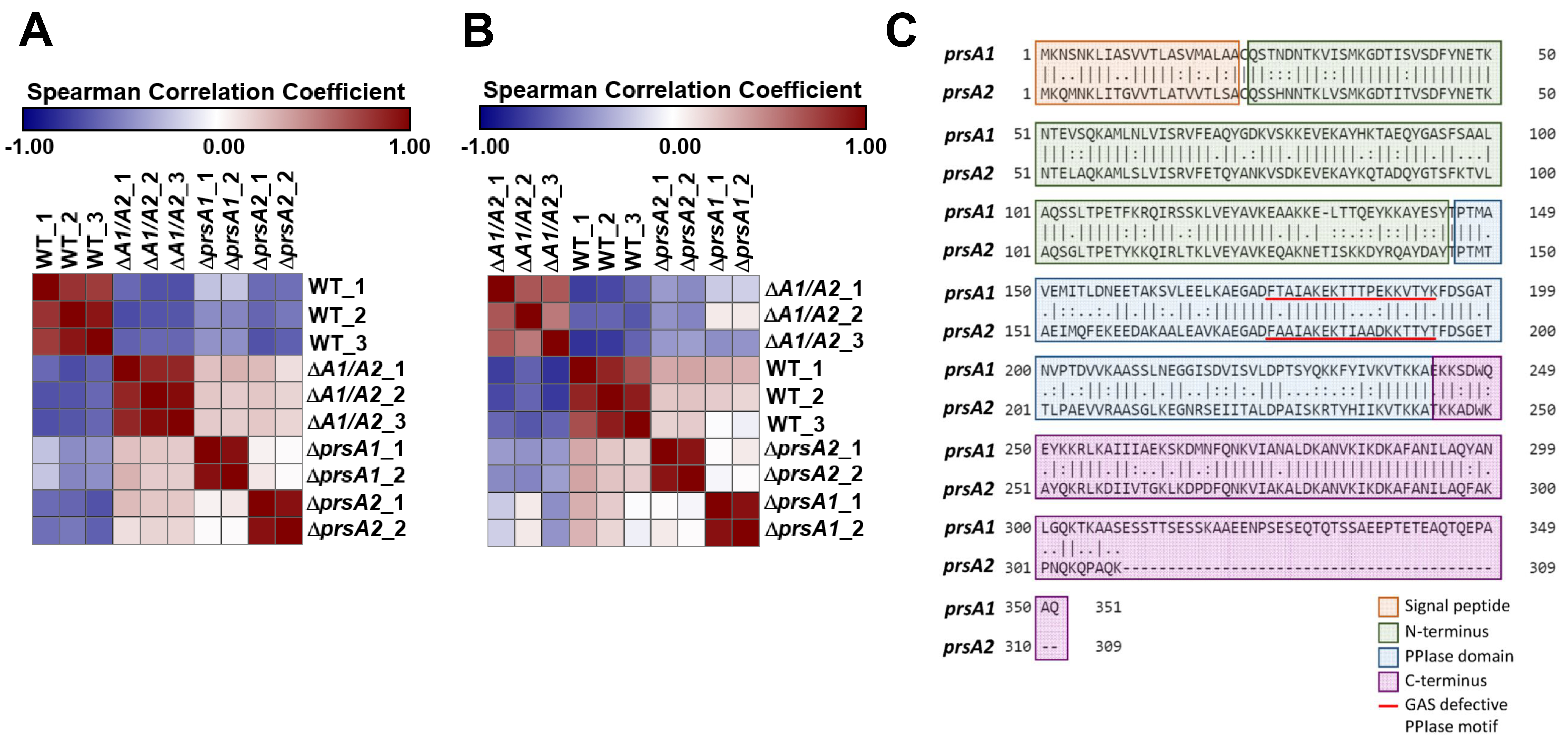

Supplement: Supplemental Material [file KVIR_A_1982501_SM3960.zip › supplementary/Fig_S2.jpg]

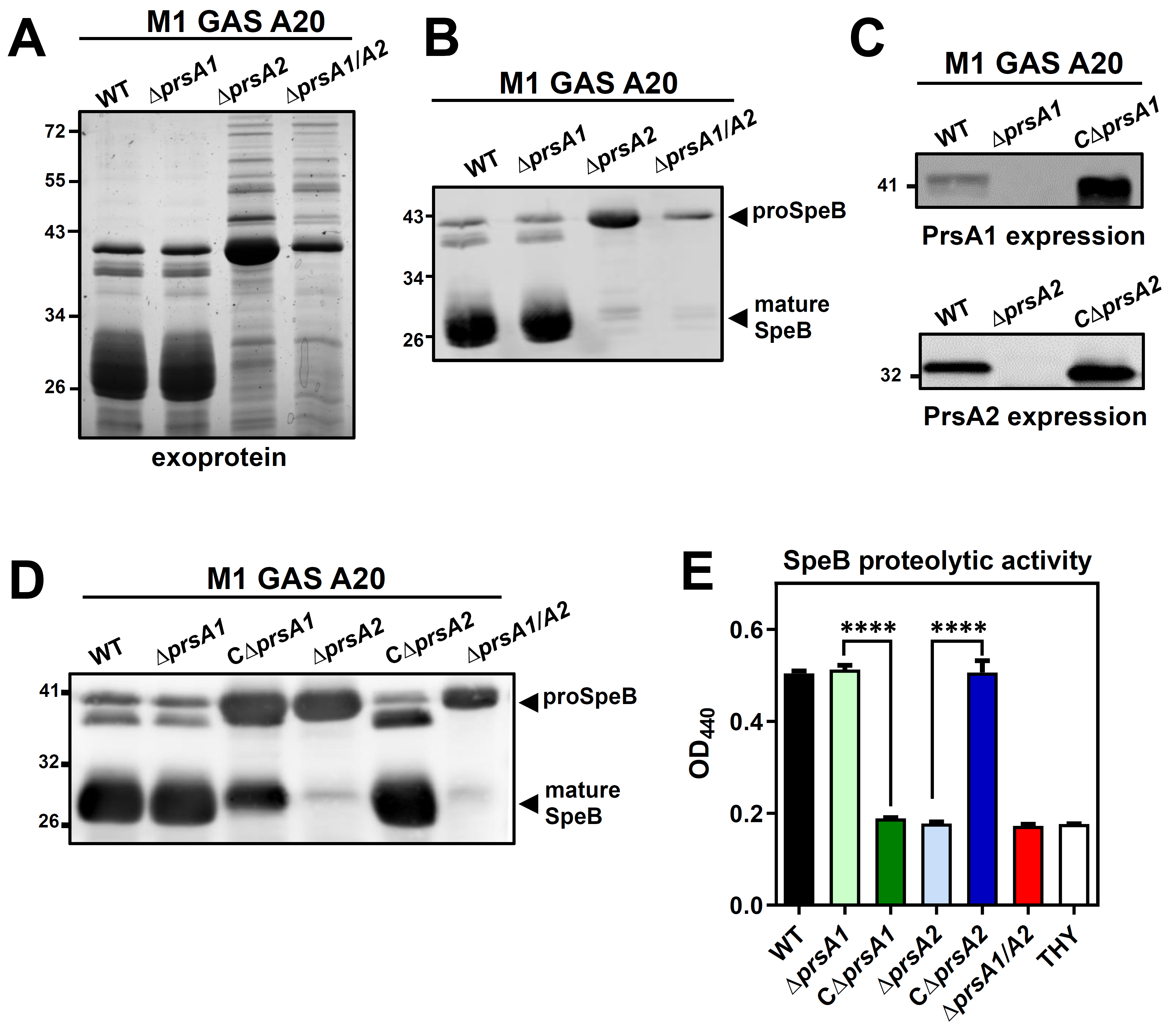

Supplement: Supplemental Material [file KVIR_A_1982501_SM3960.zip › supplementary/Fig_S3.jpg]
